# Rough backs: taxonomic value of epicuticular sculpturing in the genus *Milnesium* Doyère, 1840 (Tardigrada: Apochela)

**DOI:** 10.1038/s41598-022-10758-z

**Published:** 2022-06-14

**Authors:** Witold Morek, Karol Wałach, Łukasz Michalczyk

**Affiliations:** Department of Invertebrate Evolution, Institute of Zoology and Biomedical Research, Faculty of Biology, Jagiellonian University, Gronostajowa 9, 30-387 Kraków, Poland

**Keywords:** Zoology, Taxonomy

## Abstract

The phylum Tardigrada comprises ~ 1400 described species that inhabit a wide range of ecosystems throughout the globe. Tardigrades are generally considered taxonomically challenging due to a strongly limited number of taxonomically informative morphological traits and microscopic size. Of all tardigrade groups, the taxonomy of *Milnesium* Doyère, 1840 is particularly difficult because in comparison with most other eutardigrades, the genus lacks the taxonomically informative egg shell ornamentation and/or placoids in the muscle pharynx. Therefore, any new morphological traits that could be used in species delineation and identification are priceless. In this contribution, we review and evaluate taxonomic value of the dorsal cuticle morphology. Specifically, by means of experimental taxonomy, we demonstrate the first extreme case of ontogenetic variability in dorsal epicuticle sculpturing using a new species from Portugal, *Milnesium decorum* **sp. nov.** Furthermore, we verify the existence of dorsal gibbosities in *Milnesium reticulatum* Pilato, Binda, Lisi, 2002, the only species of the genus allegedly exhibiting these structures. Finally, we provide a diagnostic key to the *Milnesium granulatum* morphogroup.

## Introduction

The phylum Tardigrada groups microscopic eight-legged animals (usually 250–600 µm in length) belonging to the superclade Ecdysozoa^1^. These ubiquitous invertebrates inhabit almost all environments on our planet, both terrestrial and aquatic, however, to be active they require at least a water film^2^. Tardigrades, commonly named as water bears, are well-known for their ability to enter cryptobiosis and withstand harsh environmental conditions^3^. To date almost 1400 species have been formally described^4^ and this number is systematically growing. Tardigrades can feed on various food sources^5^, but only one group—the order Apochela—is considered exclusively carnivorous, as it can survive and reproduce only by hunting for rotifers, nematodes, protozoans or other tardigrades^5–7^. This order, comprising a single family Milnesiidae, is taxonomically challenging due to the low number of taxonomically meaningful morphological traits and still scarce integrative data (DNA barcodes are currently available for only one third of the described species;^8^). The family comprises four genera, three monotypic and one, *Milnesium* Doyère, 1840^9^, which groups 44 valid extant species. However, recent surveys showed that the described species constitute a small fraction of the true species diversity^8,10^.

Although there is weak correlation between taxonomically important traits and phylogeny, which prevents splitting *Milnesium* into multiple genera^10^, the genus can be divided into morphogroups that gather species exhibiting the same or similar states of morphological traits regardless of the phyletic relationships between these species. Morphogroups are useful for practical taxonomic reasons, such as constructing differential diagnoses or diagnostic keys. The two main traits used for the delineation of morphogroups in *Milnesium* are claw configuration (CC) and dorsal cuticle surface^11,12^. The CC informs about the number of points on secondary branches of claws and their position on fore- and hindlimbs, and there are currently seven recognised CC morphotypes^13,14^. In parallel, the dorsal cuticle sculpture allows for dividing *Milnesium* species into two morphogroups, the *tardigradum* and the *granulatum* morphogroup, clustering species with cuticle appearing in light microscopy as smooth or reticulated, respectively.

Although the criterion of reticulated *vs* non-reticulated cuticle seems straightforward, phase contrast microscopy (PCM) observations of cuticular surface in *Milnesium* proved to be misleading. In fact, the first described species with the epicuticular reticulum, *Milnesium granulatum* Ramazzotti, 1962^15^, was thought to have the dorsal cuticle covered with granulation. Ramazzotti^15^ interpreted bright polygons on the cuticle surface as granules (hence the species and the morphogroup name). This is surprising because granules, being thicker than the surrounding cuticle, always appear darker in PCM; thus, bright polygons must be areas where the cuticle is thinner. Indeed the first scanning electron microscope (SEM) observations of another species exhibiting the same cuticle morphotype, *Milnesium krzysztofi* Kaczmarek & Michalczyk, 2007^16^, showed that “granules” are depressions, which form a reticular system of meshes delineated by polygonal epicuticular ridges^11,16^. Soon after the *tardigradum* and the *granulatum* morphogroups were defined, *Milnesium beasleyi* Kaczmarek et al*.*, 2012^17^, a species with minute but evident and densely arranged bright spots with blurred edges was discovered. Similar spots were identified in the heterotardigrade *Barbaria madonnae* Michalczyk & Kaczmarek, 2006^18^ and SEM analysis showed that the reason why the edges cannot be focused in PCM is that they are shallow epicuticular depressions. Thus, to differentiate them from true pores that pierce the epicuticle and hence have well-defined and focusable edges, Michalczyk & Kaczmarek^18^ termed such depressions as pseudopores. Since spots in *M. beasleyi* appeared under PCM similar to spots in *B. madonnae*, Kaczmarek et al*.*^17^ interpreted them as pseudopores and classified *M. beasleyi* as a member of the *granulatum* morphogroup. However, subsequent analyses of *M. tardigradum* Doyère, 1840^9^ in high quality light and scanning electron microscopy showed that pseudopores are endocuticular channels^19^. In fact, these channels were first discovered via transmission electron microscopy (TEM) observations by Greven^20^, but they were never linked to pseudopores observed in PCM before. Probably all members of the genus have these structures, but their diameter and number differs between species, which translates to their variable visibility under light microscopy^21^. Thus, since pseudopores are not epicuticular structures, Morek et al*.*^19^ postulated to restore the original definition of the *granulatum* morphogroup, that is, restrict it to species with epicuticular reticulation. Currently, the morphogroup comprises eight species (chronologically): *M. granulatum*; *M. reticulatum* Pilato, Binda, Lisi, 2002^22^; *M. katarzynae* Kaczmarek et al*.*, 2004^23^; *M. krzysztofi*; *M. alabamae* Wallendorf & Miller, 2009^24^; *M. lagniappe* Meyer, Hinton & Dupré, 2013^25^; *M. cassandrae* Moreno-Talamantes et al*.*, 2019^26^; and *M. pacificum* Sugiura et al*.*, 2020^27^. In one of these species, *M. reticulatum*, additionally to the epicuticular reticulum, dorsal cuticular gibbosities were also described, although only a drawing and no photographic evidence of this unique trait was provided in the original description, and the species has not been recorded ever again.

In this study, we address several aspects of the cuticle morphology and its taxonomic value in the genus *Milnesium*. The description of a new species representing the *granulatum* morphogroup and a discovery of a novel cuticle morphotype provide an occasion to revise this morphogroup, including the verification of the presence of gibbosities in *M. reticulatum* via the re-examination of the type material. Finally, we construct a new diagnostic key to the discussed morphogroup.

## Materials and methods

### Sampling and specimen handling

Individuals representing the new species were extracted according to procedures described in^28^ from a moss sample collected in Portugal (see Table 1 for details). Afterwards, the extracted specimens were split into four analysis: (i) imaging and morphometry in phase-contrast light microscopy (PCM) and UV-fluorescence microscopy (UVM), (ii) imaging in scanning electron microscopy (SEM), and (iii) developmental tracking^13^ and culture establishment, and (iv) DNA extraction and sequencing. The exact number of specimens utilised for given analysis is provided in Table 1.

**Table 1:** The collection details of populations analysed in this study.

Species	Sample code	Locality	Coordinates Altitude	Sample type	Specimens analysed				GenBank accession numbers
					LCM	SEM	DEV	DNA	
*Milnesium decorum* **sp. nov**	PT.010 [**type locality**]	Portugal, Lisbon, Oeiras	38°41′24″'N 9°19′18″W 27 m asl	lichen	33	8	16	10	18S rRNA: MK484075 28S rRNA: MK483983 ITS-2: MK484010 COI: MK492287
*Milnesium* sp. nov	CO.004	Colombia, Departamento Putumayo, Sibundoy	1°8′44.3"N 76°50′43"W 2 800 m asl	moss	2	0	0	0	–

Analysis types: LCM—morphometry and imaging in PCM and UVM; DNA—DNA sequencing; SEM—imaging in SEM; DEV—developmental analysis (ontogenetic tracking).

### Microscopy, imaging and morphometry

The specimens were mounted on permanent microscope slides in Hoyer’s medium according to the method by^29^ to examine general morphology in PCM and acquire morphometric data. The measurements follow^30^, the buccal tube widths were measured according to^11,12^ and the body length was measured from the anterior to posterior margin of the body, excluding the hind legs. Pseudoplate row numbers are given according to^26^ and poorly visible pseudoplates are marked with a dotted line. The *pt* index is a ratio of a given structure to the length of the buccal tube, expressed as a percentage^31^ and in the text is given in *italics*. The number of measured specimens follow the recommendation of^32^ when it was possible, otherwise all available and properly fixed and oriented specimens were measured. Structures were measured only if their orientation was suitable. We present the joined measurements of specimens of the same CC (i.e. juveniles and adults) in a single table. The morphometric data was handled using the Apochela spreadsheet ver. 1.3. available from Tardigrada Register^33^, www.tardigrada.net. All the measurements and photographs were taken with Olympus BX53 PCM associated with Olympus DP74 digital camera (PCM). Pseudoplate arrangement was additionally determined with the UVM Nikon Eclipse 80i associated with Nikon Digital Sight DS-L2 digital camera^34^. For deep-focus structures a series of up to 22 pictures were taken and merged into one image using Corel Photo-Paint 2020. Some specimens were processed for SEM imaging according to the protocol by^28^ and examined under high vacuum with a Versa 3D DualBeam Scanning Electron Microscope at the ATOMIN facility, of the Jagiellonian University, Kraków, Poland.

In the differential diagnosis, we used two tailed Student *t*-tests to demonstrate statistically significant differences between pairs of species with slightly overlapping ranges of morphometric traits.

### Culturing and developmental tracking

Culture of the new species was established from alive specimens and eggs deposited in exuviae. The culture was incubated at rearing conditions described by^35^ with rotifers *Lecane inermis* Bryce, 1892^36^, as a food source. To test for ontogenetic variability, developmental tracking^13^ was applied.

### Genotyping

The DNA was extracted from individual specimens (see Table 1 for sample size) following the *Chelex®* 100 resin (Bio-Rad) extraction method by^37^, with modifications by^38^. Prior to the DNA extraction, the specimens were mounted on temporary water slide to check the morphology (CC). The four standard molecular markers were sequenced, three nuclear: the small ribosomal subunit (18S rRNA), large ribosomal subunit (28S rRNA), Internal Transcribed Spacer 2 (ITS-2); and one mitochondrial, Cytochrome Oxidase C subunit I (COI). The PCR protocols follow^38^, primers and PCR programmes with relevant references are listed in Table 2. The obtained chromatograms were checked manually in BioEdit ver. 7.2.5^46^. In addition the COI sequences were translated into amino acids using MEGA 7^47^ to ensure that no pseudogenes were amplified. All sequences are deposited in GenBank (accession numbers are listed in Table 1).

**Table 2:** Primers and references for specific protocols for amplification of the four DNA fragments sequenced in the study.

DNA fragment	Primer name	Primer direction	Primer sequence (5′-3′)	Primer source	PCR programme
18S rRNA	18S_Tar_Ff1	Forward	AGGCGAAACCGCGAATGGCTC	^39^	^40^
	18S_Tar_Rr1	Reverse	GCCGCAGGCTCCACTCCTGG		
28S rRNA	28S_Eutar_F	Forward	ACCCGCTGAACTTAAGCATAT	^41^	^42^
	28SR0990	Reverse	CCTTGGTCCGTGTTTCAAGAC	^42^	
ITS-2	ITS2_Eutar_Ff	Forward	GCATCGATGAAGAACGCAGC	^43^	^43^
	ITS2_Eutar_Rr	Reverse	TCCTCCGCTTATTGATATGC		
COI	LCO1490	Forward	GGTCAACAAATCATAAAGATATTGG	^44^	^11^
	HCOoutout	Reverse	GTAAATATATGRTGDGCTC	^45^	

### Comparative material

For the comparisons with the new species, we examined slides from type series of *M.* *beasleyi* Kaczmarek, Jakubowska & Michalczyk, 2012^17^ (slide no.: TR/t1/12 (holotype); TR/t1/3; TR/t1/11); *M. katarzynae* Kaczmarek, Michalczyk & Beasley, 2004^23^ (slide no.: 13/2); *M.* *krzysztofi* Kaczmarek & Michalczyk, 2007^16^ (slide no.: CR 11/2; CR 16/1; CR 467/1; CR 467/2) and *M.* *reticulatum* Pilato, Binda, Lisi, 2002^22^ (slide no.: 4855).

### Data deposition

Raw morphometric data for *M. decorum* **sp. nov.** are provided as supplementary materials (Supplementary material) and are also deposited in the Tardigrada Register^33^ under www.tardigrada.net/register/0116.htm. DNA sequences are deposited in GenBank (see Table 1 for accession numbers).

## Results and discussion

### Taxonomic account of the new species

**Figure 1:**
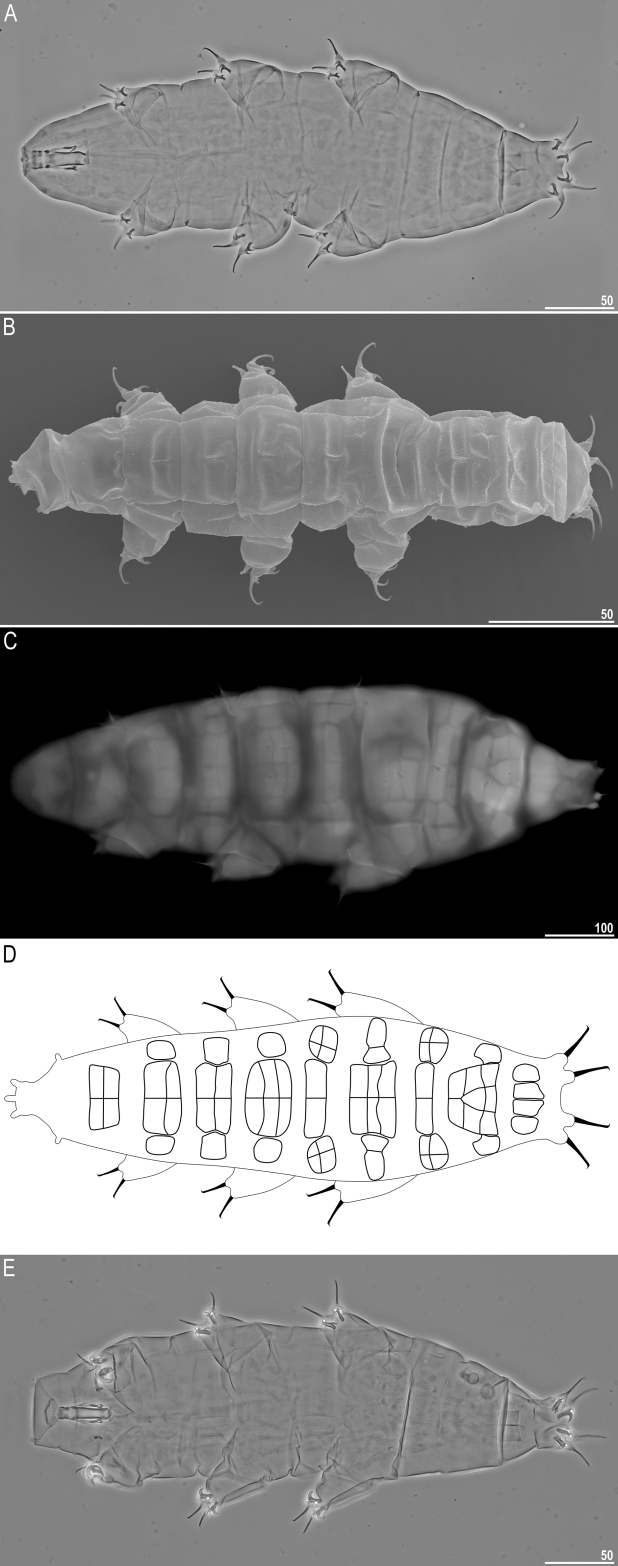
General morphology of *Milnesium decorum* **sp. nov.** (**A**) juvenile habitus, PCM (holotype, juvenile); (**B**) hatchling habitus, SEM (paratype); (**C**) adult habitus with visible pseudoplates; UVM (paratype, simplex); (**D**) pseudoplate arrangement based on the observation of holotype and paratypes in PCM, UVM and SEM (created with MS PowerPoint 2016); (**E**) mature male habitus, PCM (paratype). All scale bars in µm.

**Figure 2:**
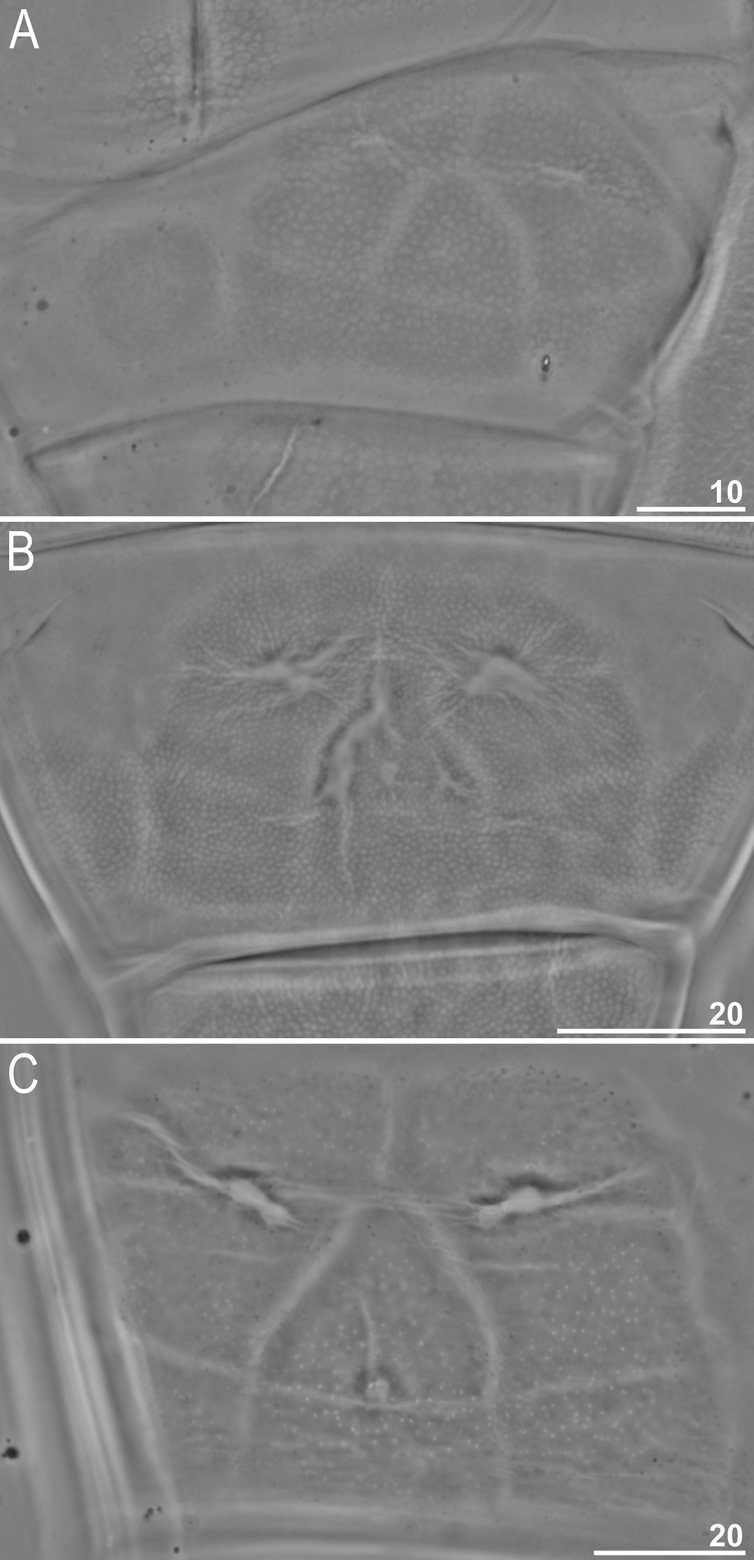
Dorsal cuticle sculpturing of *Milnesium decorum* **sp. nov.** seen in PCM: (**A**) hatchling with a clearly visible reticulation (paratype); (**B**) juvenile with a visible reticulation (holotype); (**C**) adult female with clearly visible pseudopores but unidentifiable reticulation (paratype). All scale bars in µm.

**Figure 3:**
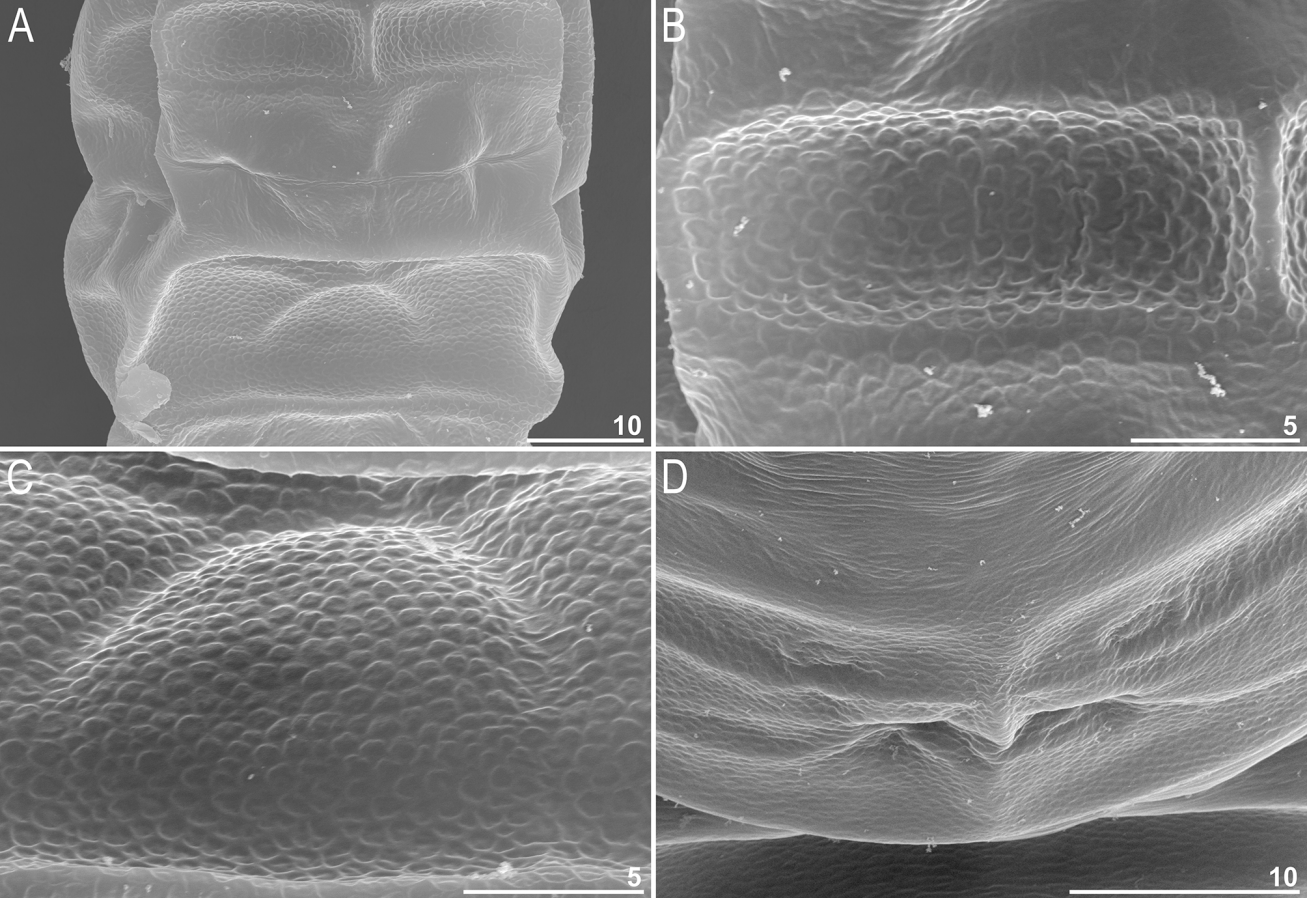
Dorsal cuticle sculpturing of *Milnesium decorum* **sp. nov.** seen in SEM: (**A**) polygonal reticulation and pseudoplates of row VII and VIII (hatchling or juvenile); (**B**) close-up on the on row VII (hatchling or juvenile); (**C**) close-up on row VIII (hatchling or juvenile); (**D**) close-up on row VIII of (adult specimen). Please note that the sculpture is formed by a polygonal reticulum with thin walls and shallow dimples and pseudoplates are concave, but because of optical illusion pseudoplates may seem convex and dimples may appear as polygonal granules [especially in (**B** and **C**)]. All scale bars in µm.

**Figure 4:**
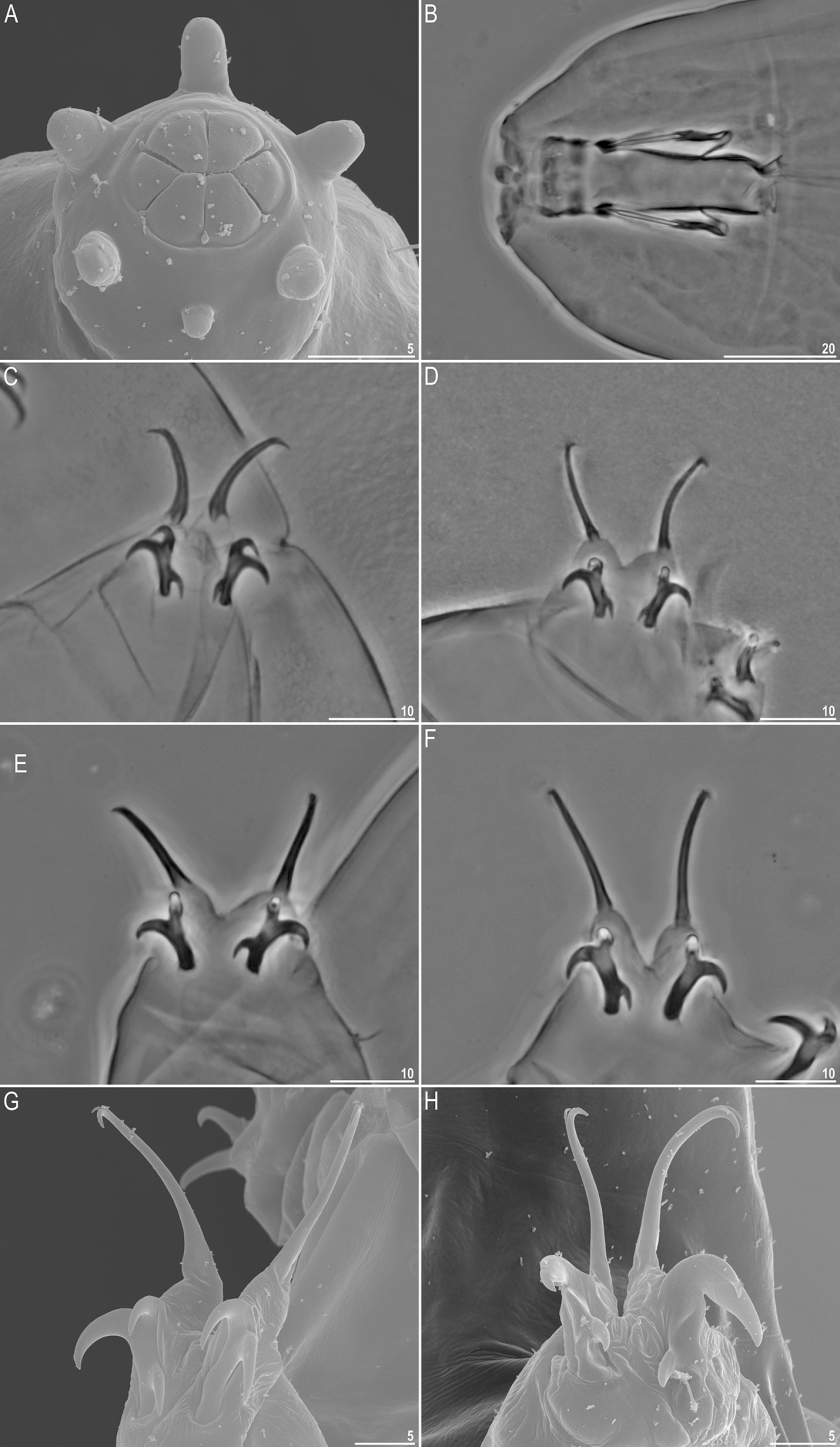
General morphology of *Milnesium decorum* **sp. nov.** (**A**) SEM photograph of mouth opening; with six, unequal in size peribuccal lamellae, so called 4 + 2 configuration (paratype); (**B**) buccal apparatus, PCM (holotype); (**C**) claws III of the hatchling, with the [3-3] CC, PCM (paratype); (**D**) claws IV of the hatchling, with the [3-3] CC, PCM (paratype). (**E**) claws III of the juvenile, with the [2-3] CC, PCM (holotype); (**F**) claws IV of the juvenile, with the [3-2] CC, PCM (holotype); (**G**) claws III of the female, with the [3-2] CC, SEM (paratype); (**H**) claws I of the male, with the secondary branches modified into rigid hooks and absent cuticular bars, SEM (paratype). All scale bars in µm.

- Phylum: Tardigrada Doyère, 1840^9^
- Class: Eutardigrada Richters, 1926^48^
- Order: Apochela Schuster et al*.*, 1980^49^
- Family: Milnesiidae Ramazzotti, 1962^15^
- Genus: *Milnesium* Doyère, 1840^9^
- ***Milnesium decorum*** **sp. nov.**
  - *Milnesium* sp. nov. 3 PT.010 in^10^
  - *Milnesium* sp. nov. PT.010 B #5 in^8^
  - Figures 1, 2, 3 and 4, Tables 3, 4 and 5; Supplementary material.

**Table 3:** Measurements (in μm) and the *pt* values of selected morphological structures of 5 females and 2 juveniles of *Milnesium decorum* **sp. nov.** from Portugal, PT.010, mounted in Hoyer’s medium. All available specimens were measured.

Character	N	Range						Mean		SD	
		µm			*pt*			µm	*pt*	µm	*pt*
Body length	7	414–783			*1357–1767*			622	*1502*	141	*146*
Peribuccal papillae length	5	6.4–10.8			*19.3–24.4*			9.1	*21.3*	1.7	*2.0*
Lateral papillae length	7	4.1–8.9			*13.4–20.1*			6.8	*16.4*	1.6	*2.0*
**Buccal tube**											
Length	7	30.5–48.8			–			41.1	–	6.7	–
Stylet support insertion point	7	19.6–28.9			*59.2–65.6*			25.8	*63.1*	3.8	*2.1*
Anterior width	7	11.0–23.0			*33.7–51.9*			17.0	*40.8*	4.8	*6.7*
Standard width	7	9.4–19.6			*30.4–44.2*			14.8	*35.6*	4.1	*5.2*
Posterior width	7	9.3–20.0			*28.0–44.7*			14.6	*34.9*	4.5	*6.3*
Standard width/length ratio	7	30%–44%			–			36%	–	5%	–
Posterior/anterior width ratio	7	79%–96%			–			86%	–	6%	–
**Claw 1 heights**											
External primary branch	7	13.2–21.7			*38.0–45.2*			17.7	*43.2*	3.1	*2.6*
External base + secondary branch	7	10.3–16.2			*30.6–36.7*			13.8	*33.6*	2.2	*2.3*
External branches length ratio	7	74%–82%			–			78%	–	3%	–
Internal primary branch	7	13.3–21.1			*35.9–44.6*			17.1	*41.8*	2.8	*3.0*
Internal base + secondary branch	7	9.9–15.8			*28.0–34.9*			13.2	*32.2*	2.2	*2.4*
Internal spur	5	4.2–5.5			*11.3–13.8*			5.2	*12.4*	0.6	*0.9*
Internal branches length ratio	7	74%–80%			–			77%	–	2%	–
**Claw 2 heights**											
External primary branch	7	15.5–22.5			*40.4–50.8*			18.8	*46.0*	2.8	*4.0*
External base + secondary branch	6	9.7–16.5			*30.6–36.1*			14.1	*33.2*	2.6	*2.4*
External branches length ratio	6	63%–82%			–			73%	–	6%	–
Internal primary branch	6	14.8–19.9			*40.2–48.5*			17.5	*44.4*	2.0	*3.5*
Internal base + secondary branch	5	9.7–15.8			*30.4–35.7*			13.6	*32.4*	2.5	*2.2*
Internal spur	7	4.2–6.9			*12.2–18.7*			5.8	*14.3*	0.9	*2.2*
Internal branches length ratio	4	66%–79%			–			75%	–	6%	–
**Claw 3 heights**											
External primary branch	7	14.2–21.0			*41.6–49.4*			18.6	*45.4*	2.6	*2.9*
External base + secondary branch	7	10.3–16.6			*30.9–36.2*			14.0	*34.1*	2.3	*1.9*
External branches length ratio	7	69%–82%			–			75%	–	4%	–
Internal primary branch	6	15.5–19.9			*40.8–46.7*			18.3	*42.9*	1.6	*2.1*
Internal base + secondary branch	7	10.4–15.6			*29.2–35.5*			13.5	*33.0*	2.1	*2.3*
Internal spur	5	4.3–6.4			*12.2–15.7*			5.4	*13.7*	0.8	*1.3*
Internal branches length ratio	6	70%–82%			–			76%	–	4%	–
**Claw 4 heights**											
Anterior primary branch	7	16.1–26.0			*49.3–58.1*			21.9	*53.5*	3.5	*2.9*
Anterior base + secondary branch	7	11.3–17.7			*34.2–40.0*			15.2	*37.0*	2.4	*2.4*
Anterior spur	5	4.5–6.5			*10.6–16.9*			5.4	*13.9*	0.8	*2.3*
Anterior branches length ratio	7	64%–77%			–			69%	–	4%	–
Posterior primary branch	7	18.0–27.1			*52.2–61.7*			23.3	*57.1*	3.4	*3.4*
Posterior base + secondary branch	7	11.1–18.4			*34.8–41.3*			15.5	*37.7*	2.7	*2.2*
Posterior branches length ratio	7	61%–73%			–			66%	–	5%	–

**Table 4:** Measurements (in μm) and the *pt* values of selected morphological structures of 5 males of *Milnesium decorum* **sp. nov.** from Portugal, PT.010, mounted in Hoyer’s medium. All available specimens were measured.

Character	N	RANGE						MEAN		SD	
		µm			*pt*			µm	*pt*	µm	*pt*
Body length	1	410–410			*1459–1459*			410	*1459*	?	*?*
Peribuccal papillae length	1	3.9–3.9			*12.1–12.1*			3.9	*12.1*	?	*?*
Lateral papillae length	1	4.4–4.4			*15.7–15.7*			4.4	*15.7*	?	*?*
**Buccal tube**											
Length	5	27.6–32.3			–			29.6	–	1.9	–
Stylet support insertion point	5	17.4–20.7			*61.5–65.2*			18.8	*63.5*	1.3	*1.6*
Anterior width	5	8.0–9.9			*27.9–33.1*			9.1	*30.8*	0.7	*2.5*
Standard width	5	6.6–7.7			*23.5–25.1*			7.2	*24.4*	0.5	*0.7*
Posterior width	5	6.9–7.7			*22.3–26.1*			7.3	*24.8*	0.3	*1.5*
Standard width/length ratio	5	23%–25%			–			24%	–	1%	–
Posterior/anterior width ratio	5	78%–86%			–			81%	–	3%	–
**Claw 1 heights**											
External primary branch	4	15.0–16.6			*50.8–55.5*			15.5	*53.8*	0.7	*2.2*
External base + secondary branch	5	11.6–12.8			*39.6–44.5*			12.4	*42.0*	0.5	*1.9*
External branches length ratio	4	76%–83%			–			79%	–	3%	–
Internal primary branch	2	15.1–15.2			*53.7–55.1*			15.2	*54.4*	0.1	*0.9*
Internal base + secondary branch	5	12.1–13.3			*39.3–45.6*			12.6	*42.9*	0.5	*2.6*
Internal spur	1	5.0–5.0			*15.5–15.5*			5.0	*15.5*	?	*?*
Internal branches length ratio	2	80%–85%			–			82%	–	4%	–
**Claw 2 heights**											
External primary branch	5	16.0–18.9			*54.5–63.0*			17.5	*59.2*	1.3	*3.4*
External base + secondary branch	5	11.2–12.3			*37.5–43.1*			11.8	*40.1*	0.5	*2.3*
External branches length ratio	5	65%–71%			–			68%	–	2%	–
Internal primary branch	4	15.6–18.0			*52.6–60.2*			16.7	*56.8*	1.0	*3.2*
Internal base + secondary branch	5	10.8–13.0			*38.5–42.7*			11.9	*40.4*	0.8	*1.7*
Internal spur	5	3.0–5.9			*10.7–21.4*			5.0	*16.8*	1.2	*4.0*
Internal branches length ratio	4	69%–76%			–			72%	–	4%	–
**Claw 3 heights**											
External primary branch	4	16.2–18.3			*54.2*–65.1			17.4	*60.4*	1.0	*4.6*
External base + secondary branch	5	10.2–12.6			*34.1*–42.7			11.6	*39.3*	0.9	*4.3*
External branches length ratio	4	63%–70%			–			67%	–	3%	–
Internal primary branch	4	16.3–18.6			*53.9*–63.3			17.5	*59.6*	1.0	*4.2*
Internal base + secondary branch	4	10.9–12.4			*38.4*–41.6			11.8	*39.9*	0.6	*1.4*
Internal spur	3	5.1–6.0			*15.8*–20.3			5.6	*18.7*	0.5	*2.5*
Internal branches length ratio	4	65%–71%			–			67%	–	3%	–
**Claw 4 heights**											
Anterior primary branch	2	21.7–22.9			*72.6–81.5*			22.3	*77.0*	0.8	*6.3*
Anterior base + secondary branch	5	12.1–13.6			*40.5–46.3*			12.8	*43.4*	0.6	*2.2*
Anterior branches length ratio	2	57%–60%			–			58%	–	2%	–
Posterior primary branch	2	19.0–19.3			*64.5–67.6*			19.2	*66.1*	0.2	*2.2*
Posterior base + secondary branch	3	11.6–12.5			*38.8–44.1*			12.2	*41.6*	0.5	*2.7*
Posterior spur	1	5.6–5.6			*19.9–19.9*			5.6	*19.9*	?	*?*
Posterior branches length ratio	2	60%–65%			–			63%	–	4%	–

**Table 5:** Measurements (in μm) and the *pt* values of selected morphological structures of 9 hatchlings of *Milnesium decorum* **sp. nov.** from Portugal, PT.010, mounted in Hoyer’s medium. All available specimens were measured.

Character	N	RANGE						MEAN		SD	
		µm			*pt*			µm	*pt*	µm	*pt*
Body length	8	265–322			*1085–1314*			295	*1206*	19	*78*
Peribuccal papillae length	5	3.4–4.3			*13.9–18.0*			3.9	*16.0*	0.5	*1.9*
Lateral papillae length	4	3.1–4.5			*12.9–18.1*			3.8	*15.3*	0.6	*2.2*
**Buccal tube**											
Length	9	23.6–25.6			–			24.3	–	0.6	–
Stylet support insertion point	9	15.7–16.8			*64.1–69.2*			16.3	*67.1*	0.3	*1.7*
Anterior width	9	7.1–8.3			*28.5–35.0*			7.6	*31.3*	0.5	*2.3*
Standard width	9	6.1–7.1			*25.5–28.7*			6.6	*27.1*	0.3	*1.0*
Posterior width	9	6.2–7.0			*25.3–29.5*			6.6	*27.3*	0.3	*1.4*
Standard width/length ratio	9	26%–29%			–			27%	–	1%	–
Posterior/anterior width ratio	9	83%–93%			–			87%	–	4%	–
**Claw 1 heights**											
External primary branch	8	11.5–13.1			*47.5–54.0*			12.3	*50.5*	0.6	*2.1*
External base + secondary branch	8	8.3–9.5			*34.6–37.6*			8.8	*36.1*	0.4	*1.0*
External spur	6	1.7–3.1			*7.1–12.5*			2.3	*9.3*	0.5	*1.8*
External branches length ratio	7	68%–75%			–			71%	–	2%	–
Internal primary branch	7	10.6–13.1			*42.7–52.0*			11.6	*47.5*	1.0	*3.3*
Internal base + secondary branch	9	8.0–9.4			*33.1–39.7*			8.7	*35.8*	0.4	*2.0*
Internal spur	8	2.1–3.4			*8.5–14.4*			2.9	*12.1*	0.5	*2.1*
Internal branches length ratio	7	69%–84%			–			77%	–	6%	–
**Claw 2 heights**											
External primary branch	8	11.4–14.6			*47.1–57.0*			13.1	*53.5*	0.9	*3.1*
External base + secondary branch	8	8.3–9.8			*34.6–39.3*			9.1	*37.4*	0.5	*1.4*
External spur	6	2.2–3.1			*9.2–12.1*			2.6	*10.7*	0.3	*1.2*
External branches length ratio	7	67%–71%			–			68%	–	2%	–
Internal primary branch	8	11.0–13.9			*44.4–54.3*			12.1	*49.8*	0.9	*3.3*
Internal base + secondary branch	7	8.5–9.2			*34.7––37.3*			8.8	*36.2*	0.3	*0.9*
Internal spur	7	2.3–4.0			*9.3–16.9*			3.2	*13.1*	0.6	*2.8*
Internal branches length ratio	6	66%–83%			–			73%	–	6%	–
**Claw 3 heights**											
External primary branch	9	12.3–14.6			*51.3–57.7*			13.3	*54.5*	0.8	*2.2*
External base + secondary branch	9	8.6–10.1			*35.8–39.5*			9.2	*37.7*	0.4	*1.3*
External spur	4	2.3–3.2			*9.0–12.9*			2.7	*10.7*	0.4	*1.7*
External branches length ratio	9	63%–73%			–			69%	–	3%	–
Internal primary branch	6	12.3–13.6			*49.6–55.3*			12.9	*52.9*	0.5	*1.9*
Internal base + secondary branch	7	8.6–9.7			*35.5–39.6*			9.1	*37.3*	0.4	*1.4*
Internal spur	8	2.4–3.6			*9.4–15.3*			3.0	*12.4*	0.4	*2.0*
Internal branches length ratio	5	69%–76%			–			72%	–	3%	–
**Claw 4 heights**											
Anterior primary branch	7	13.1–15.5			*54.6–62.5*			14.1	*58.2*	0.8	*3.4*
Anterior base + secondary branch	5	8.4–9.5			*34.7–39.7*			8.9	*36.8*	0.4	*2.1*
Anterior spur	5	2.8–3.6			*11.7–15.2*			3.1	*12.9*	0.3	*1.4*
Anterior branches length ratio	5	62%–66%			–			64%	–	2%	–
Posterior primary branch	8	14.0–16.9			*57.9–66.9*			15.5	*63.4*	1.0	*3.4*
Posterior base + secondary branch	5	8.8–9.6			*37.1–38.7*			9.1	*37.6*	0.3	*0.6*
Posterior spur	4	1.9–3.1			*7.9–12.9*			2.6	*10.5*	0.5	*2.1*
Posterior branches length ratio	5	56%–64%			–			61%	–	4%	–

### Integrative description

#### Mature females (from the third instar onwards; morphometrics and holotype measurements in Table 3)

Moderate length *Milnesium* species, up to 783 µm (Fig. 1), yellow. Eyes present in all living individuals and in the majority of Hoyer-fixed specimens (9/11; 82%). The dorsal cuticle covered with reticulum, which is clearly visible on pseudoplates (Figs. 1B, 2) and weakly developed in the remaining areas of the dorsum (Figs. 2–3). In larger specimens (4^th^ + instars), the reticulation may be poorly visible in PCM (Fig. 2C). This species is characterised by numerous pseudoplates (Fig. 1C–D) arranged in nine transverse rows, which are clearly visible both in PCM and UVM: (I) a single trapezoid pseudoplate (divided into four equal rectangular portions); (II) a large central rectangular pseudoplate (divided into four rectangular portions, with the two anterior rectangles being larger) + two lateral oval pseudoplates; (III) central rectangular pseudoplate (divided into four equal rectangular portions, concave laterally) + lateral square pseudoplates with protuberance matching the concave sides of the central plate; (IV) large roundish central pseudoplate (divided into six equal rectangular portions) + roundish lateral pseudoplates; (V) a central rectangular pseudoplate (divided longitudinally into two equal rectangles) + two lateral roundish pseudoplates (divided into four unequal rectangular portions); (VI) a large central rectangular pseudoplate (divided into six equal rectangular portions) + two lateral elongated pseudoplates with curvy edges (divided longitudinally into two unequal portions); (VII) a central rectangular pseudoplate (divided longitudinally into two equal rectangles) + two lateral rectangular pseudoplates (divided into four unequal rectangular portions); (VIII) the largest, most complex, trapezoid pseudoplate (divided into eight parts: a central triangle and seven quadrangles) + two roundish lateral pseudoplates with small projections; (IX) four pseudoplates arranged transversally (internal trapezoid and the lateral roundish).

Mouth opening surrounded with six short peribuccal papillae (with the ventral one being the smallest) and six triangular peribuccal lamellae of unequal size (with the two lateral lamellae significantly smaller, i.e. the 4+2 configuration; Fig. 4A). The lamellae configuration is unambiguously visible only in SEM. Two short lateral cephalic papillae present. Buccal tube cylindrical and of moderate width (Fig. 4B).

Typical *Milnesium* claws. Primary branches with tiny accessory points visible both in PCM and SEM (Fig. 4E–G). Internal and anterior secondary branches equipped with the basal spur, i.e. with a [2-3]-[3-2] CC (Fig. 4E–G). Cuticular bars under claws I–III absent in the majority of specimens (9/11; 82%), and faint and barely visible when present.

#### Mature males (from the third instar onwards; morphometrics in Table 4)

In the sample only single male was found (preserved on SEM stub) but the culture yielded additional 10 specimens. Smaller than females (Fig. 1E), with narrower buccal tube and with modified first pairs of claws into rigid hooks (Fig. 4H), as in all other *Milnesium* species. Eyes present in living animals, but absent in 7/10 (70%) of Hoyer-fixed specimens. Cuticular bars under claws always absent (these are the first *Milnesium* males reported to be lacking cuticular bars).

#### Juveniles (second instar, morphometrics, including holotype measurements in Table 3)

Morphologically similar to adult females but with a better developed dorsal reticulation and more weakly outlined dorsal pseudoplates (Fig. 2B). Eyes present in living animals but absent in both Hoyer-fixed specimens. Cuticular bars absent. Other qualitative traits as in adult females.

#### Hatchlings (first instar, morphometrics in Table 5)

Morphologically similar to juveniles but with a better developed dorsal reticulation and more weakly outlined dorsal pseudoplates (Fig. 2A). All secondary branches equipped with spurs, i.e. with a [3-3]-[3-3] CC (Fig. 4C,D). Eyes present in living animals but absent in all 11 Hoyer-fixed specimens. Cuticular bars absent. Other qualitative traits as in adult females.

#### Ontogenetic variability

*Milnesium decorum* **sp. nov.** undergoes developmental changes in two key taxonomic traits, cuticular sculpturing and CC. The dorsal cuticle sculpturing becomes less clear with every consecutive instar. Specifically, under PCM, it is most pronounced in hatchlings, slightly less developed in juveniles, and it is very weakly outlined in adults or even not visible at all in large adult females. Under SEM, the reticulum also fades with subsequent moults, but it is detectable in all life stages (Fig. 3). The CC changes from [3-3]-[3-3] in hatchlings to [2-3]-[3-2] in juveniles, i.e. the species is characterised by early negative CC change.

#### Eggs

Smooth, oval, slightly yellowish; deposited in exuviae; up to 4 in a single clutch were found in the culture.

#### DNA markers and phylogenetic position

All four markers were represented by single haplotypes and their sequences were of the following lengths: 18S rRNA: 1055 bp (GenBank: MK484075), 28S rRNA: 801 bp (MK483983), ITS-2: 489 bp (MK484010), and COI: 559 bp (MK492287). The new species represents the Palaearctic clade A sensu^8,10^.

#### Type locality

38°41′24″N, 9°19′18″W, 27 m asl: Portugal, Lisbon District, Oeiras, Conde Oeiras Lane; lichen on a tree branch; city.

#### Etymology

The name of the species highlights the pronounced dorsal cuticle sculpturing in hatchlings and juveniles, composed of epicuticular reticulation and endocuticular pseudoplates. From Latin *decōrus* = decorated, beautiful.

#### Type repositories

The series consists of the holotype (juvenile, PT.010.39) and 40 paratypes, 32 on microscope slides (PT.010.39–65) and 8 on an SEM stub (10.09). All slides and the SEM stub are deposited at the Institute of Zoology and Biomedical Research, Jagiellonian University, Gronostajowa 9, 30–387 Kraków, Poland.

#### Remarks

The species was accompanied with a *Milnesium* sp. from the *almatyense* complex (*Milnesium* sp. #5 PT.010 A in^8^), which was much more abundant in the sample. All of the 26 eggs of *M. decorum* **sp. nov.** incubated in the laboratory culture hatched, and nine hatchlings and seven juveniles were fixed on microscope slides. All remaining ten juveniles kept in the culture eclosed into males. Thus, with no adult females obtained in the laboratory, the culture was terminated. As a result, most of the type series consists of hatchlings and males. Because of that, the type series contains small number of mature females, which are in addition poorly to moderately preserved, thus as a result we designated a juvenile as the holotype.

#### Adult and juvenile phenotypic differential diagnosis

*Milnesium decorum* **sp. nov.** is one of the 13 described species of *Milnesium* characterised by a [2-3]-[3-2] adult CC, and can be differentiated specifically from:

- *M. barbadosense* Meyer & Hinton, 2012^50^; *M.* *pseudotardigradum* Surmacz, Morek & Michalczyk, 2019^51^; *M.* *reductum* Tumanov, 2006^30^; *M.* *tardigradum* Doyère, 1840^9^; *M.* *tetralamellatum* Pilato & Binda, 1991^52^ and *M. vorax* Pilato, Sabella & Lisi, 2016^53^; by the well-visible nine rows of pseudoplates *vs* invisible or poorly visible just an outline of single pseudoplate (row VIII) in the remaining species.
- *M. beasleyi* Kaczmarek, Jakubowska & Michalczyk, 2012^17^, known only from type locality in Turkey, by the relatively shorter lateral papillae (*13.4–20.1*, mean *16.4*, N = 7 in the new species *vs 19.6–23.7*, mean *21.5*, N = 5 in *M. beasleyi*; *t* = 5.446, df = 8, p < 0.001), the cuticular sculpture (well-visible reticulum in PCM in juveniles *vs* pseudoporous cuticle lacking reticulum in *M. beasleyi*).
- *M. lagniappe* Meyer, Hinton & Dupré, 2013^25^; recorded from the United States, by a relatively more anterior stylet support insertion point (SSIP) (*59.2–65.6* in the new species *vs 69.7–73.4* in *M. lagniappe*) and by a relatively shorter primary claw branch IV (*49.3–61.7* in the new species *vs 62.9–76.6* in *M.* *lagniappe*).
- *M. krzysztofi* Kaczmarek & Michalczyk, 2007^16^, reported from Costa Rica and Peru^54^, by the appearance of the dorsal reticulum (thin-walled in the new species (see Fig. 2 herein) *vs* thick-walled in *M. krzysztofi*, Figs. 6–11 in^16^ and Fig. 7B,C herein) and by relatively longer spurs of all claws (*10.6–18.7* in the new species *vs 8.0–10.8* in *M. krzysztofi*).
- *M. cassandrae* Moreno-Talamantes et al*.*, 2019^26^, found only in several localities in Mexico, by a relatively narrower standard buccal tube width (*25.5–28.7* in the new species *vs 41.6–67.2* in *M. cassandrae*) and by a different direction of the ontogenetic CC change (negative in the new species *vs* positive in *M. cassandrae*).
- *M. pacificum* Sugiura, Minato, Matsumoto & Suzuki, 2020^27^, reported from three islands of Japan, by the relatively longer spurs on claws I and II (*11.3–13.8* on claws I and *12.2–18.7* on claws II in the new species *vs 5.3–11.7* on claws I and *6.0–12.2* on claws II in *M. pacificum*) and by a different pattern of the ontogenetic CC change (negative in the new species *vs* positive in *M. pacificum*).
- *M. reticulatum* Pilato, Binda & Lisi, 2002^22^, known only from the Seychelles*,* by a relatively more anterior stylet support insertion point (SSIP) (*59.2–65.6* in the new species *vs 68.5–69.8* in *M. reticulatum*) and by a relatively longer primary claw branch IV (*49.3–61.7* in the new species *vs 37.9–44.3* in *M.* *reticulatum*).

#### Hatchling phenotypic differential diagnosis

*Milnesium decorum* **sp. nov.** hatchlings differ from the two described species with a [3-3]-[3-3] CC and reticulated cuticle:

- *M*. *alabamae* Wallendorf & Miller, 2009^24^, recorded only from USA (Alabama), by a relatively narrower standard buccal tube width (*25.5–28.7* in the new species *vs 29.5–44.0* in *M. alabamae*).
- *M. granulatum* Ramazzotti, 1962^15^, known only from Chile (the only confirmed record), by a relatively narrower standard buccal tube width (*25.5–28.7* in the new species *vs 46.3* in *M. granulatum* paratype; morphometrics from^11^).

#### Genetic differential diagnosis

The ranges of uncorrected p-distances between the new species and available sequences for other congeners are as follows:

- **18S rRNA**: 1.0–3.2% (2.2% on average), with the most similar being *M.* *dornensis* Ciobanu, Roszkowska & Kaczmarek, 2015^55^ (MK484071^10^), and the least similar being unidentified species from Australia (MK484082/“*Milnesium* sp. nov. 13 AU.052 B” in^10^).
- **28S rRNA**: 2.2–10.5% (7.2% on average), with the most similar being an unidentified species from Canary Island, Spain (MK483995/“*Milnesium* sp. nov. 2 ES.012” in^10^) and the least similar being an unidentified species from the Philippines (MK484004/“*Milnesium* sp. nov. 10 PH.014” in^10^).
- **ITS-2:** 8.4–20.6% (13.0% on average), with the most similar being an unidentified species from Canary Island, Spain (MK484020/“*Milnesium* sp. nov. 2 ES.012” in^10^) and the least similar being an unidentified species from Australia (MK484015 and MK484018/“*Milnesium* sp. nov. 11 AU. 52 A and AU.105” in^10^).
- **COI:** 11.2–22.3% (16.0% on average), with the most similar being *M.* *dornensis* (MK484071^19^), and the least similar being an unidentified species from Australia (MK492295/“*Milnesium* sp. nov. 11 AU.105” in^10^).

### Amendment of the *Milnesium reticulatum* Pilato, Binda & Lisi, 2002 description

The re-examination of four *M. reticulatum* paratypes under PCM confirmed that the dorsal cuticle is covered with a distinct and robust (thick-walled) reticulum, similar to that in *M. krzysztofi* (compare Fig. 7 herein and Fig. 6 in^16^). However, in contrast to the original description^22^, which states that “7 transversal rows of not very prominent gibbosities are present [on the dorsum]”, we saw no gibbosities on the dorsal cuticle of *M.* *reticulatum*. Instead, we observed poorly outlined pseudoplates, which were misinterpreted as gibbosities in the original description (confirmed by personal communication with Oscar Lisi). Due to poor preservation of the examined specimens, the determination of the exact pseudoplate arrangement was not possible.

Given that the gibbosities alone were sufficient to differentiate *M. reticulatum* from all other known congeners, but now this traits has been invalidated, the validity of similar species published after the description of *M. reticulatum* needs to be verified. In addition to *M. reticulatum*, there are only five other known *Milnesium* species with a [2-3]-[3-2] CC and reticulated dorsal cuticle: *M. cassandrae*, *M. decorum* **sp. nov.**, *M. krzysztofi, M. lagniappe* and *M. pacificum*. These species all differ from *M. reticulatum* by relative morphometric traits, specifically by the *pt* of the SSIP (*68.5–69.8* in *M. reticulatum vs 58.7–67.6* in *M. cassandrae*, *59.2–65.6* in *M. decorum* **sp. nov.**, and *63.3–67.3* in *M. krzysztofi*) and by the *pt* of the anterior primary claw branch height (*37.9–39.7* in *M. reticulatum vs 62.9–74.0* in *M. lagniappe*, *41.6–65.9* in *M. pacificum* and *49.3–61.7* in *M. decorum* **sp. nov.**). Therefore, the amendment of *M. reticulatum* description does not entail any changes in the taxonomic status of other congeners.

The original description of *M. reticulatum* highlights the importance of providing the readers with raw data, such as photomicrographs, as this is the only way the scientific community may widely and at any time evaluate the interpretation and conclusions laid out by the authors of the original contribution (the re-examination of specimens is not always possible and much more difficult than accessing raw data provided in the article, supplementary materials or in open data repositories). In^22^, all images, including the dorsum and the alleged gibbosities, are in the form of drawings, thus the reader is presented only with an interpretation. It has been demonstrated that morphological interpretations may vary considerably between researchers^56^ and the original description of *M. reticulatum* is a striking example of this phenomenon. Another misinterpretation of *Milnesium* morphology was recently exposed by^57^ who showed that the alleged three spines on the dorsum of the invalid now “*Milnesium tardigradum trispinosa*”^58^ were, in fact, folds of the cloacal cuticle. Have there been photographs of these structures provided in the original contributions, the scientific community most likely would have falsified them earlier. Thus, although drawings can be a useful addition to photomicrographs in tardigrade taxonomy, they should not be the only mean of morphological illustration.

### Cuticle sculpturing in the genus *Milnesium*

**Figure 5:**
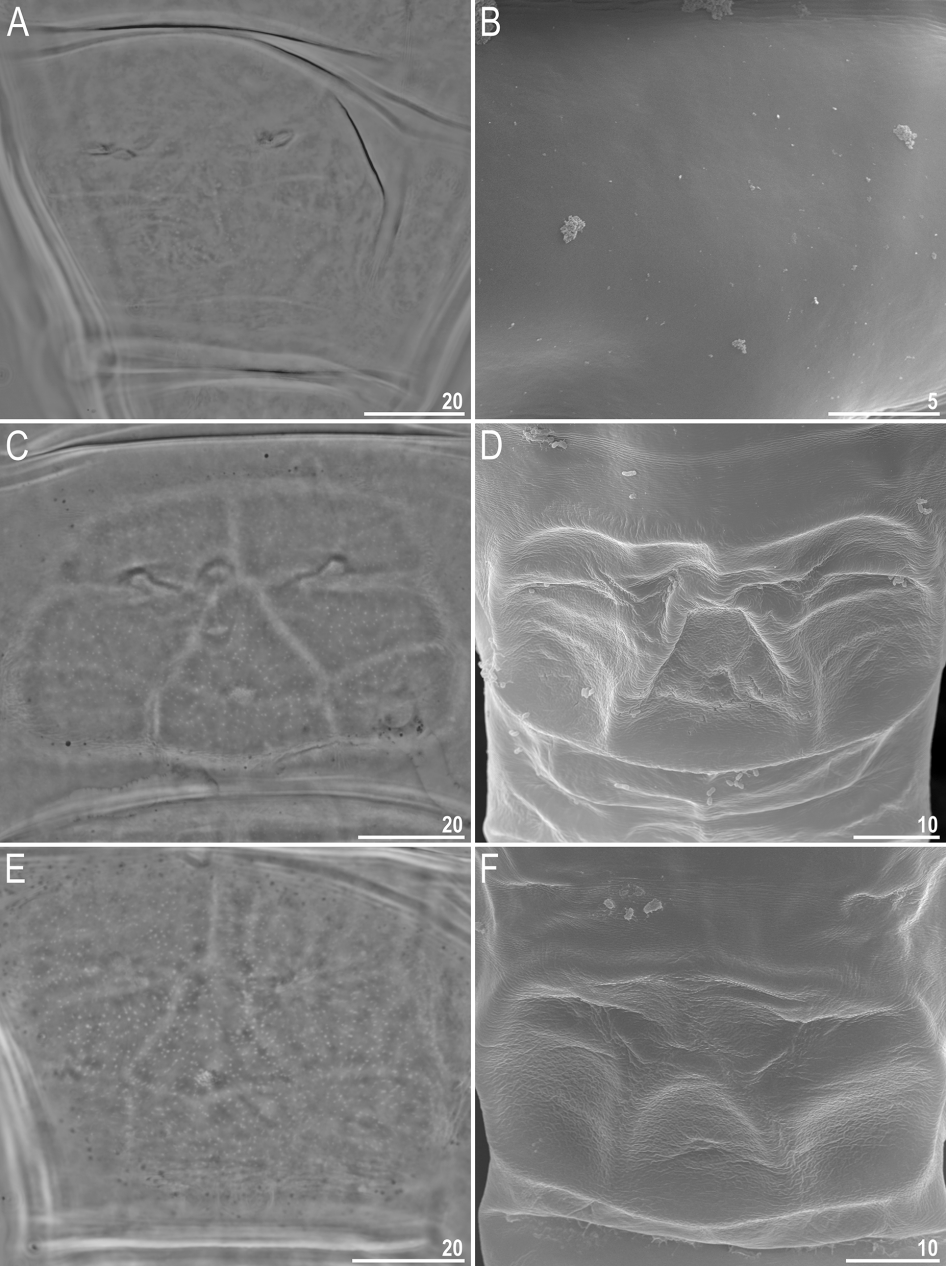
Examples of cuticle morphology of the *Milnesium tardigradum* morphogroup. (**A**) dorsal cuticle of *M. tardigradum*, with barely visible pseudopores, PCM; (**B**) smooth dorsal cuticle of *M. tardigradum* SEM; (**C**) dorsal cuticle of *M. variefidum*, with well-visible but tiny pseudopores, PCM (holotype); (**D**) dorsal cuticle of *M. variefidum* with wrinkles on pseudoplate VIII, SEM; (**E**) dorsal cuticle of *M.﻿ beasleyi*, with large and well-visible pseudopores, PCM (paratype); (**F**) dorsal cuticle of *M. berladnicorum*, with fine reticulum-like sculpturing on pseudoplate VIII, SEM. All scale bars in µm.

**Figure 6:**
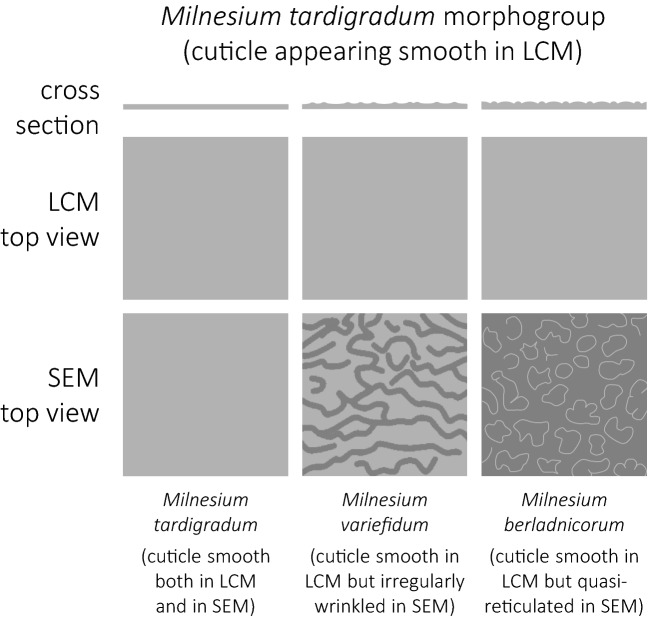
Schematic drawings showing a different appearance of “smooth” cuticle in the *Milnesium tardigradum* morphogroup under LCM (top panel) and SEM (bottom panel). Whereas cuticle is truly smooth in *M tardigradum* (left panel), it exhibits fine irregular wrinkles *M. variefidum* (middle panel) and an irregular quasi-reticulum with small bumps in *M. berladnicorum* (right panel) that are below LCM resolution.

**Figure 7:**
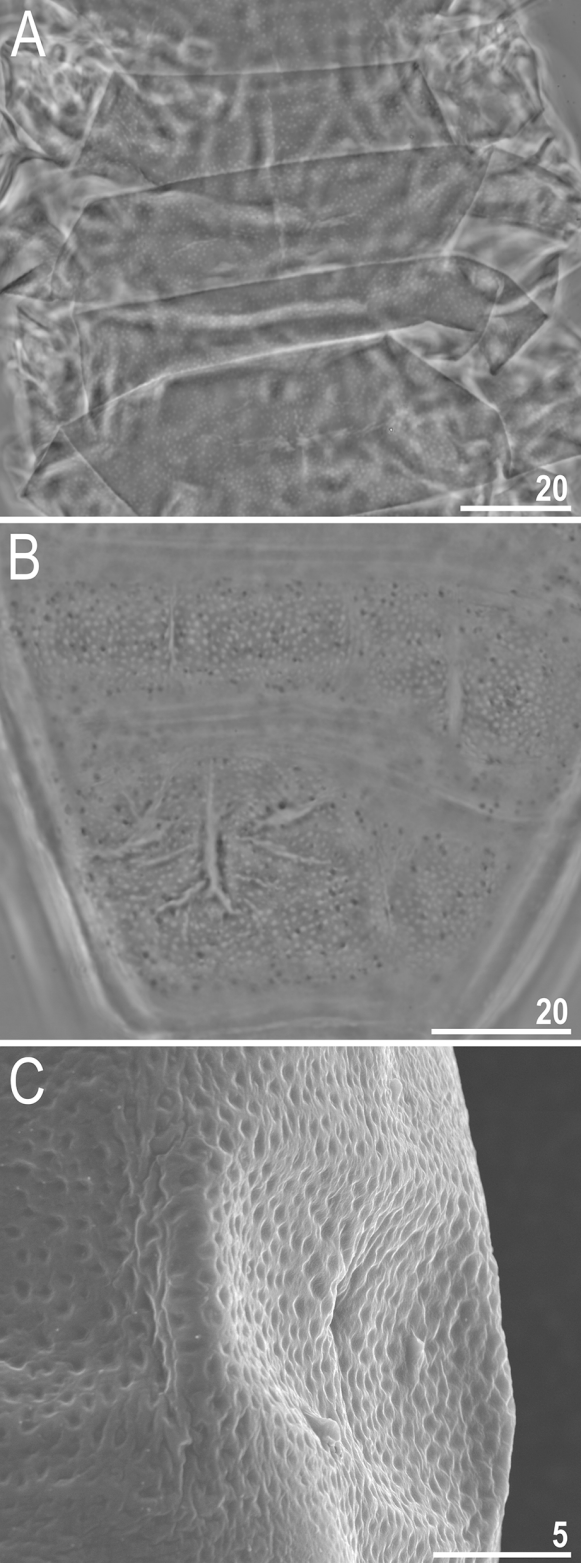
Cuticle morphology of the *Milnesium granulatum* morphogroup. (**A**) dorsal cuticle of *M. reticulatum*, PCM; in the microphotograph the reticulation is clearly visible but the gibbosities are absent; (**B**) dorsal cuticle of *M. krzysztofi*, PCM; (**C**) dorsolateral cuticle of *M. krzysztofi*, SEM. All scale bars in µm.

**Figure 8:**
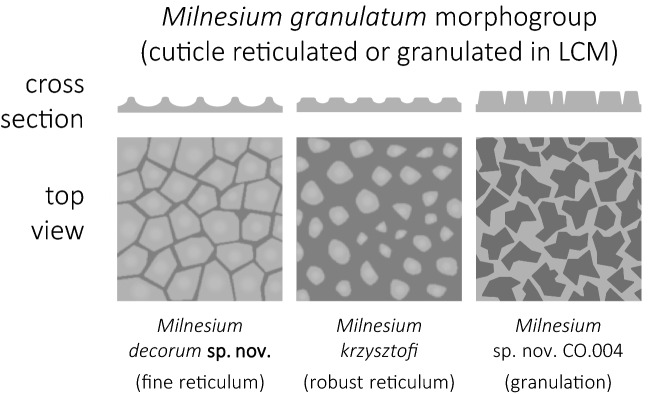
Schematic drawings showing different types of epicuticular sculpturing in the *Milnesium granulatum* morphogroup (based on LCM and SEM observations). The top panel shows cross sections through the cuticle surface, whereas the bottom panel shows top views on the cuticle surface (darker grey indicates thicker cuticle/elevated surface). The left panel shows a fine reticulum with thin walls and wide meshes (*M. decorum* **sp. nov.**), the middle panel shows a robust reticulum with thick walls and small meshes (*M. krzysztofi* and the great majority of species of the *granulatum* morphogroup), and the right panel shows granulation (*Milnesium* sp. nov. CO.004).

**Figure 9:**
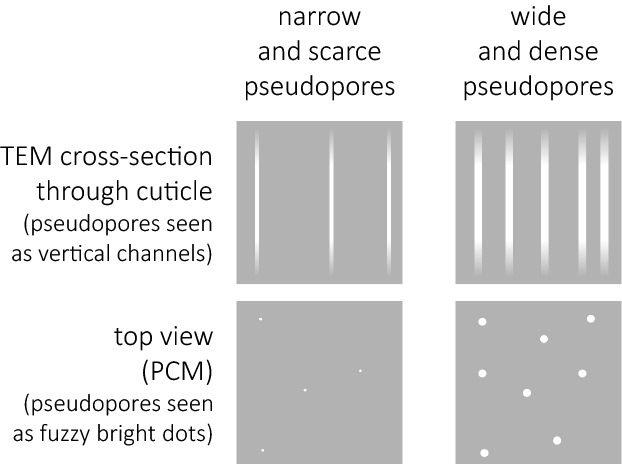
Schematic drawings showing variation in the diameter and density of pseudopores in *Milnesium* cuticle (based on LCM observations and TEM photomicrographs by^20^). Small and scarce pseudopores (left panel) are harder to see in LCM than large and densely arranged pseudopores (right panel). The top drawings show cross sections through cuticle, whereas the bottom drawings illustrate a top-down view.

Among the formally described *Milnesium* species, the dorsal cuticle surface can be divided into two main morphotypes when observed under the light contrast microscope (LCM): smooth (e.g. *M. tardigradum*, *M. beasleyi* or *M. variefidum* Morek, Gąsiorek, Stec, Blagden, Michalczyk, 2016^13^) and reticulated (e.g. *M. granulatum*, *M. krzysztofi* or *M. decorum* **sp. nov.**). These two morphogroups have been named after the first described species exhibiting given morphotype, i.e. the *tardigradum* (Figs. 5 and 6) and the *granulatum* (Figs. 7 and 8) morphogroup^11^). Furthermore, these can be split into subgroups. Specifically, some species of the *tardigradum* morphogroup have weakly (e.g. *M. tardigradum*; Figs. 5A and 9), moderately (e.g. *M. variefidum*; Fig. 5C) or strongly (e.g. *M. beasleyi*; Figs. 5E and 9) developed pseudopores. In individuals of the *granulatum* morphogroup, in which the reticulum is clearly visible, pseudopores are not always easy to observe, but the reticulum may be robust (thick-walled with small meshes, as in *M. krzysztofi*; Figs. 7B and 8) or fine (thin-walled with large meshes, as in *M. decorum* **sp. nov.**; Figs. 2 and 8). Although the variability within this trait requires further research, as intermediate morphotypes may be present, *M. decorum* **sp. nov.** is the only known species with such fine reticulum.

When observed in SEM, cuticle of species representing the *granulatum* morphogroup appears similar as in LCM (e.g. compare Figs. 2B and 3C). However, the cuticle of species of the *tardigradum* morphogroup, although appears smooth under LCM, is not always smooth in SEM. For example, whereas it is indeed smooth in *M. tardigradum* (Figs. 5B and 6), it is finely and more or less regularly wrinkled in *M. variefidum* (Figs. 5D and 6) or covered with irregular quasi-reticulum (interlaced ridges with bumps filling the meshes) in *M. berladnicorum* Ciobanu, Zawierucha, Moglan, Kaczmarek, 2014^61^ (Figs. 5F and 6). Since only a fraction of *Milnesium* species have been imaged in SEM, the taxonomic value of fine sculpturing identifiable only in SEM is yet to be evaluated when more data are available. Nevertheless, the term “smooth cuticle” has to be used carefully, always with the reference to the type of microscope that was used to make the distinction.

**Figure 10:**
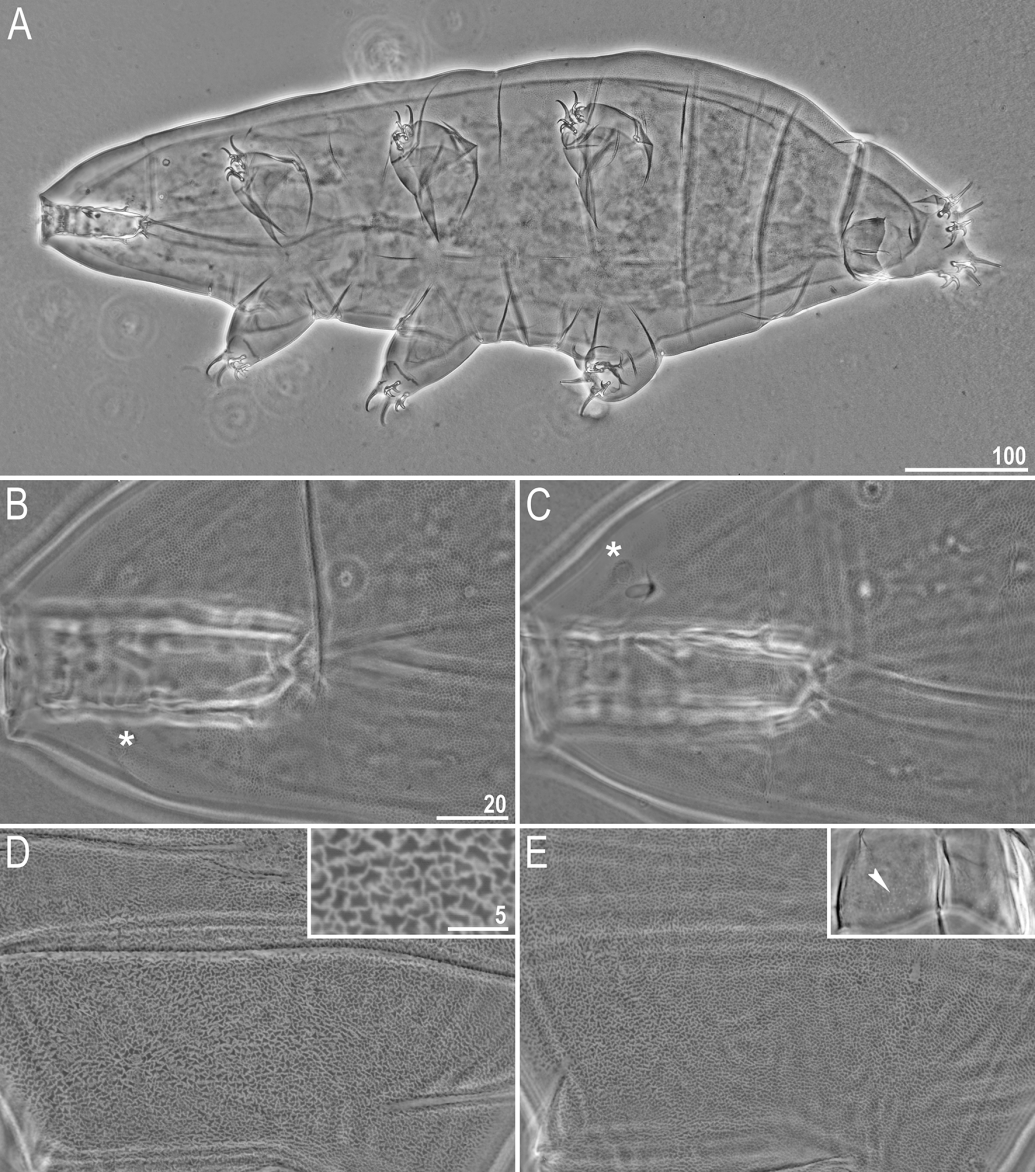
*Milnesium* sp. nov. CO.004 from Colombia, with irregular polygonal granulation visible on the entire body in PCM. (**A**) habitus, ventral view, with granulation visible in the caudal part of the body; (**B**, **C**) the same part of the head in dorsal and ventral view, respectively (asterisks indicate small smooth areas of the cuticle close to the lateral papillae); (**D**, **E**) the same fragment of the caudal part of the body between legs III and IV, in the dorsal and ventral view, respectively; the insert on D shows a magnified fragment of the dorsal cuticle sculpturing; the insert on E shows the pseudopores visible on the cloaca. The scale bar in μm; scale the same on the (**B**–**E**).

However, as more new species in the genus are uncovered, new types of cuticular sculpturing may be revealed. In fact, we have found such a new morphotype represented by an undescribed species collected in Colombia (*Milnesium* sp. nov. CO.004; Table 1; Figs. 8 and 10). This species is characterised by a genuine granulation present on the entire body, including the ventral side, which has never been reported in any *Milnesium* species before. The granulation is slightly larger on the dorsum than on the ventral side and in the caudal part compared to the cephalic part of the body, but all granules are evident in LCM (Fig. 10). The granules are in the shape of irregular polygons, most often concave and with 7–10 edges (Fig. 10D). Besides granulation, we observed pseudopores, but only in the cloacal cuticle. (Fig. 10E, insert). Even though this is clearly a new species, we refrain from describing it as a new taxon because of the lack of associated DNA sequences and the low number of available specimens (N = 2). The small sample size prevents the assessment of intraspecific variability and the exclusion of morphological aberration as the explanation for this extraordinary phenotype. Moreover, if there are more species exhibiting this type of sculpturing, describing this Colombian species without genetic data could make it difficult to delineate these hypothetical similar species, creating a potential taxonomic impediment that we have already seen too many times in the history of tardigrade research (e.g. see^59^). In other words, we are of the opinion that the species should be described only when more individuals are found and their DNA is sequenced (see also^13^).

**Figure 11:**
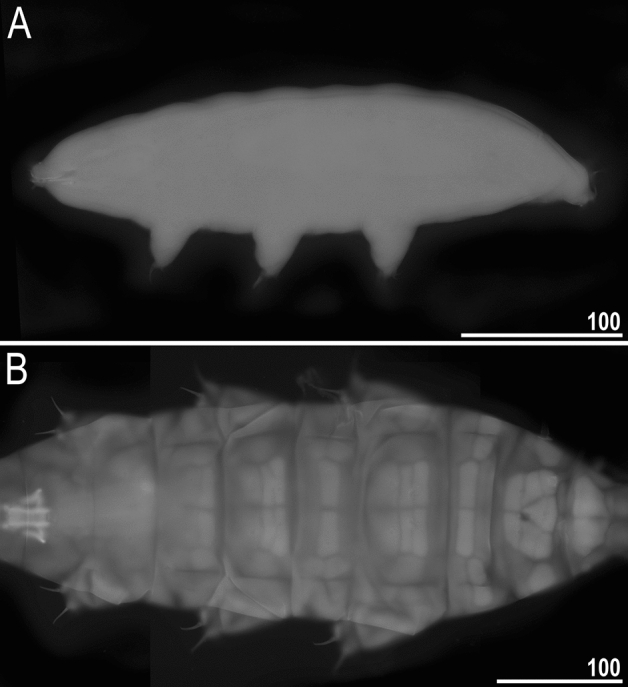
Evidence that not all *Milnesium* species exhibit pseudoplates: (**A**) *M. tardigradum* adult (no pseudoplates are visible); (**B**) *M. variefidum* adult (pseudoplates are clearly visible, especially in the caudal part of the body); both mounted in the same medium and observed under the same UVM and with the same camera. All scale bars in µm.

In addition to epicuticular sculpturing and endocuticular pseudopores, some *Milnesium* species also exhibit endo- or sub-cuticular areas of thicker cuticle described and termed as pseudoplates independently by^13^ and^60^; however, they have been noted before although without naming them (e.g.^17,61^). Moreover,^13^ suggested that the number, shape and arrangement of these structures could possibly be used for species delineation and identification, but this view was questioned by^26^, who hypothesised that pseudoplates do not exhibit variation within the genus and therefore should not be used as a taxonomic trait. However, our extensive analysis of numerous species, some represented by multiple populations, under both PCM and UVM showed that there are species, such as *M. tardigradum* (Fig. 11), that never exhibit pseudoplates. Thus, although it needs to be thoroughly tested whether in species with pseudoplates the shape and arrangement of these structures may be subject to interspecific variation, the presence *vs* absence of pseudoplates appears to be a valid discriminative taxonomic trait.

### Ontogenetic variability in dorsal cuticle in the genus *Milnesium*

In the great majority of *Milnesium* species, for which ontogeny has been described, cuticle appears similar or the same both in sexually immature and mature instars, except for endocuticular pseudopores and pseudoplates that are usually absent or less developed and therefore more difficult to identify in hatchlings and juveniles than in adults (see *M. variefidum*^13^ and *M. tardigradum* in^19^). However, there are two species in which ontogenetic variability in the epicuticular sculpturing has been observed: *M. pacificum*^27^ and *M. decorum* **sp. nov.** (the present study). In both these species, the reticulation is most developed in hatchlings and it becomes weaker with each consecutive instar, but the differences between the life stages are more pronounced in the latter taxon. Given that ontogeny has been investigated only in a small fraction of species, more research is needed to draw more general conclusions about the frequency and direction of developmental variability in cuticle appearance. Although the analysis of ontogenetic variability makes species descriptions more difficult, on the other hand, it provides extra characters for species delineation and identification in this taxonomically challenging genus.

### Diagnostic key to the *Milnesium granulatum* morphogroup

The *granulatum* morphogroup is defined here as a polyphyletic group of *Milnesium* species in which cuticular reticulation on the dorsal cuticle is visible under LCM at least in one life stage (i.e. in hatchlings [H] and/or juveniles [J] and/or adults [A]). The morphogroup currently comprises 9 formally described species (20% of the known *Milnesium* species). Morphometric data in the key refer to sexually immature and mature individuals collectively.

- **1.** Claw configuration [2–2]-[2–2] ........................................................................................... (2)
**–.** Different claw configuration .............................................................................................. (4)
- **2.** The *pt* values of the stylet support insertion point above *73* ................... *M. katarzynae* [H*]
**–.** The *pt* values of the stylet support insertion point below *73* ............................................. (3)
- **3.** Buccal tube below 23.0 µm ....................................................................... *M. cassandrae *[H]
**–.** Buccal tube above 23.0 µm ........................................................................ *M. pacificum* [H]
- **4.** Claw configuration [2–3]-[3–2] ........................................................................................... (5)
**–.** Claw configuration [3–3]-[3–3] ........................................................................................... (9)
- **5.** Four peribuccal lamellae present ........................................................................................ (6)
**–.** Six peribuccal lamellae present .......................................................................................... (7)
- **6.** The *pt* values of the anterior primary branches below *45* ................... *M. reticulatum* [H + J*]
**–.** The *pt* values of the anterior primary branches at least *55* ................. *M. lagniappe* [H + J + A]
- **7**. The reticulum thin-walled and very regular ................................ *M. decorum* **sp. nov.** [J + A]
**–.** The reticulum thick-walled and mostly irregular ................................................................ (8)
- **8.** The *pt* values of the buccal tube standard width at least *47* .................. *M. cassandrae* [J + A]
**–.** The *pt* values of the buccal tube standard width lower than *47* ..............................................
- ........................................................................... *M. krzysztofi* [J + A*]and *M. pacificum* [J + A]^†^
- **9.** Primary branches without accessory points ....................................... *M. alabamae* [H + J + A]
**–.** Primary branches with accessory points .......................................................................... (10)
- **10.** The *pt* values of the buccal tube standard width higher than *35* ........... *M. granulatum* [A*]
**–.** The *pt* values of the buccal tube standard width lower than *35* ..... *M. decorum* **sp. nov. **[H]
*The original description of the species is most likely based only on the indicated life stage(s)
- ^†^The two species are phenotypically indistinguishable based on original descriptions

## Conclusions and future directions

We have integratively described the 45th species of the genus *Milnesium*. The new species, *M. decorum* **sp. nov.**, represents the *granulatum* morphogroup and is the most striking example of ontogenetic variability in epicuticular sculpturing to date. We also amended the description of *M. reticulatum*, demonstrating that gibbosities are not present in any of the known *Milnesium* species. Moreover, our study showed that more research is needed to clarify the types of fine epicuticular sculpturing that are identifiable only under SEM, but appear as smooth cuticle under LCM. Further studies should also address the taxonomic value of pseudoplate number, shape and arrangement. Finally, the lack of evidence for phenotypic differences between *M. krzysztofi* and *M. pacificum*, noted when constructing the diagnostic key, calls for an integrative redescription of the senior species and is a reminder that utmost care must be takes when differentiating new and described *Milnesium* species.

## Supplementary Information

Supplementary Information.
